# Diversity analysis of agronomic and nutritional traits of hybrid offspring of forage bermudagrass

**DOI:** 10.3389/fpls.2023.1165707

**Published:** 2023-06-28

**Authors:** Jianmin Chen, Shugao Fan, Shuang Li, Xinyu Cui, Erick Amombo, Mingxia Ji, Xiaoyan Liu, Jinmin Fu

**Affiliations:** Coastal Salinity Tolerant Grass Engineering and Technology Research Center, Ludong University, Yantai, China

**Keywords:** bermudagrass, diversity analysis, variety comparison, forage quality, crossbred population

## Abstract

Because of its excellent stress resistance and forage quality, the forage bermudagrass hybrid population had attracted the attention of scientific researchers in recent years. Studying its diversity could promote the breeding of desirable varieties. The variability in agronomic traits including fresh weight, dry weight, ash content, crude protein content, crude fat, phosphorus content, and relative feed value for 56 bermudagrass was investigated using Wrangler as an experimental reference. Grey correlation analysis and cluster analysis were employed to screen bermudagrass with high yield and superior quality. WCF-34 had the highest 2-year fresh weight (109,773.3 kg/ha), WCF-37 had the highest 2-year dry weight (31,951.6 kg/ha), WCF-24 had the lowest Ash content (7.46%), WCF-26 had the highest crude protein content (16.27%), WCF-27 had the highest curde fat content (3.58%), WCF-13 had the highest P content (0.45%), and WCF-42 had the highest relative feed value (95.32). Combining the results of grey relational analysis and cluster analysis, WCF-42, WCF-34, WCF-38, WCF-37, and WCF-40 were selected as high-quality bermudagrass. Through comprehensive analysis of the agronomic characters of bermudagrass, five bermudagrass were selected, the outcomes of this study would provide a theoretical basis for the breeding and genetic enhancement of bermudagrass.

## 1 Introduction

*Cynodon dactylon* is a warm-season perennial plant of the Gramineae family that is extensively distributed in China (Wang et al., 2021). Bermudagrass grows fast; has a high yield, high disease resistance, numerous leaves, and good palatability; and is preferred for cattle. Hence, it is extensively used as fodder. Under proper management, forage bermudagrass may be cut three to four times per year, with a hay yield of 2,250–3,000 kg/ha (Yi et al., 2008). The crude protein and acid detergent fiber contents may achieve the forage quality of second-grade alfalfa, an excellent forage crop. Past studies have shown that Chinese bermudagrass has rich genetic diversity, which provides material and theoretical support for bermudagrass genotype improvement (Liu, 2013; Zheng et al., 2015; Li et al., 2017; Deng et al., 2021).

Wild bermudagrass has a significant disadvantage as a fodder grass when compared to a mature commercial hybrid (Li et al., 2021). The United States began to utilize wild bermudagrass resources for cross-breeding in the early 20th century, developing multiple excellent commercial cultivars and establishing large-scale grass varieties breeding and seed production specialty. However, more than half of China’s high-quality forage seeds are imported. Therefore, the screening of high-quality forage varieties is an effective means to solve the current problem. Cross-breeding is used as a common means of forage improvement. In this paper, 48 F1 bermudagrass genotypes were obtained by crossing the well-established commercial variety Wrangler with the wild bermudagrass CD21 in HuBei as the parents; seven common bermudagrass varieties 48 F1 bermudagrass and Wrangler were used as experimental materials to compare and analyze yield and quality traits, investigate the agronomic and nutritional traits diversity of 56 bermudagrass materials in yield and feed quality, screen excellent bermudagrass, and enrich the germplasm resource.

## 2 Materials and methods

### 2.1 Plant materials

A total of 56 bermudagrass samples were studied, 48 of which were F1 offspring of an early Wrangler–CD21 hybridization. Two groups of Wrangler and CD21 (composed of several clones or individual plants) were collected from the United States and Hubei province and were tetraploid species. Seed propagation and then asexual propagation were performed. Each variety for this experiment was composed of 25 offspring (i.e., 25 hybrid seeds).

The F1 hybrid bermudagrass seeds were collected in 2019 and planted in a greenhouse tray the following October. The seedlings were put in the nourishing bowl when they had four leaves. Each nutrient plate contained one bermudagrass seedling. They were planted in May 2020, with 25 plants in each plot, 48 of them numbered WCF1–WCF48; seven samples of common bermudagrass were numbered R101, R102, R103, R104, R105, R106, and R107, and the planting area was 1.5 m × 1.5 m, with Wrangler as the control variety, and three replicates were set up for the control species.

### 2.2 Overview of the test site

The test site was 127 m above sea level at the Coastal Salinity Tolerant Grass Engineering and Technology Research Center of Ludong University, with geographical coordinates of 121.53°E, 37.52°N, and a mid-latitude area. The average annual precipitation was 829.3 mm, and the testing period was from July 2021 to September 2022. **Table 1** displays the weather data during this time period. The soil at the location has a pH of 6.58, a total N content of 0.07%, an ammonia nitrogen content of 5.56 ppm, a nitrate nitrogen content of 5.6 ppm, a total P content of 0.09%, and a available P content of 61.98 ppm.

**Table 1:** Meteorological data of Yantai from 2021 to 2022.

Items	2021				2022			
	July	August	September	October	June	July	August	September
Average high temperature (°C)	29	28	26	20	29	29	29	25
Average low temperature (°C)	23	22	19	9	19	23	22	17
Total precipitation (mm)	10.5	110.3	1.9	38.2	14.3	3.6	36.2	234.9

### 2.3 Experimental design

The area of the plot was 1.5 m × 1.5 m, and the interval between the plot was 0.5 m. The yield was repeatedly investigated for 2 years. Protective lines were set up around the test plot. Output was measured once a month and four times a year. The agronomic traits of bermudagrass were determined, and excellent bermudagrass was selected based on grey relational analysis (GRA) and cluster analysis.

### 2.4 Measurements

On 21 July, 21 August, 25 September, and 24 October 2021, the bermudagrass was clipped at a height of 5 cm. On 15 June, 18 July, 23 August, and 23 September 2022, the bermudagrass was clipped at a height of 3 cm. After cutting, the mass was weighed using an electronic balance.

Each fresh sample was put in an envelope and dried to constant weight at 105°C for 30 minutes. Then, it was further dried at 75°C until a constant weight was achieved. Then, the total weight of each bermudagrass was measured according to the formula total dry weight = sample dry weight/sample fresh weight × the fresh weight of the sample, and the fresh to dry ratio was calculated (fresh to dry ratio = total fresh weight/total dry weight). The dried samples were used to determine the content of the crude ash (Ash) (Zeng and Cao, 2017), crude protein (CP) (Wang, 2021), crude fat (CF) (Wei et al., 2004), phosphorus content, neutral detergent fiber (NDF), acid detergent fiber (ADF) (Yang et al., 2012), and relative feed value (RFV) (Hong et al., 2011). The direct ash method was used to determine the crude ash content, while the Kjeldahl nitrogen determination instrument was used to determine the crude protein content. The crude fat was determined using the hot extraction oil gravimetric method by the fat assay instrument, the total P content was determined by the automatic chemical analyzer, and the neutral detergent fiber and acid detergent fiber were determined using the neutral detergent method and acid detergent method, using the following formulas: DDM (%DM) = 88.9 − 0.779 × ADF (%DM), DMI (%BW) = 120/NDF (%DM), and RFV = DMI × DDM/1.29 to calculate the relative feed value.

### 2.5 Data analysis

Microsoft Excel 2010 was used to calculate the mean value, standard deviation, and coefficient of variation of fresh weight, dry weight, and quality traits of bermudagrass. The index was divided into 10 grades, the first grade was Xi< (X − 2 σ), while the 10th level was Xi > (X + 2 σ), the difference between each stage in the middle is 0.5σ. The genetic diversity index (Shannon Weaver diversity index, H′) was calculated as H′ = −Σ(Pi)ln(Pi), where X represents the average number of each indicator, σ represents the index’s standard deviation, and Pi represents the percentage of materials with grade I in the total. SPSS 16.0 was used for statistical analysis, single-factor analysis of variance (ANOVA) was used for the analysis of significance among components, two-factor analysis of variance was used to analyze the interaction between genotype and month, and cluster analysis was conducted using the Ward method.

According to the grey correlation theory, the quality characters (Ash, CP, CF, P content, RFV, 2-year dry weight, and fresh/dry ratio) of the tested varieties were regarded as a grey system, and the optimal value of each character was selected to build an ideal variety X_0_, took the sequence composed of the characters of the tested bermudagrass as the comparison sequence, and calculate the correlation degree. The greater the correlation degree, the better the comprehensive characters. WCF-1–WCF-48 were numbered X_1_–X_48_. R102, R104, R106, R107, R101, R103, R105, and Wrangler were numbered X_49_–X_56_. The calculations of the correlation coefficient and correlation degree were as follows (Zeng et al., 2020).


(1) $${\text{ϵ}}_{i}(\text{k})=\frac{\min_{i}\min_{k}\left|{\text{X}}_{0}(\text{k})-{\text{X}}_{i}(k)\right|+\text{ρ}\max_{i}\max_{k}\left|{\text{X}}_{0}(k)-{\text{X}}_{i}(k)\right|}{\left|{\text{X}}_{0}(\text{k})-{\text{X}}_{i}(k)\right|+\text{ρ}\max_{i}\max_{k}\left|{\text{X}}_{0}(\text{k})-{\text{X}}_{i}(\text{k})\right|}$$

(2) $$R_{i}=\frac{1}{\text{n}}\sum_{k=1}^{n}{\text{ϵ}}_{i}(\text{k}),$$

(3) $$W_{k}=\frac{R_{i}}{\sum R_{j}},$$

(4) $${R^{\prime }}_{i}=\sum_{k=1}^{n}W_{k}\epsilon_{i}(\text{k}),$$

Where |X_0_(k) − X*_i_* (k)| was the absolute difference between X_0_ series and X*_i_* series at k, min*_i_* min*_k_* |X_0_(k) − X*_i_* (k)| was the second level minimum difference, max*_i_* max*_k_* |X_0_(k) − X*_i_* (k)| was the second level maximum difference, ρ was the distinguishing coefficient, where the value was considered to be 0.5, *W_k_* was the weight, and *R*′*_i_* was the weighted relevance.

## 3 Results

### 3.1 Variations of dry and fresh weights

**Table 2** showed the fresh weight variations of 56 bermudagrass components in 2021. The coefficient of variation of fresh weight of bermudagrass ranged between 16.3% and 25.6%. The greatest coefficient of variation of fresh weight of grass in October was 25.6%, while the smallest coefficient of variation of fresh weight of grass in September was 16.3%.

**Table 2:** Comparison of biomass variation of 56 forage bermudagrass samples in 2021.

Items	Fresh weight (kg/ha)					Dry weight (kg/ha)									
	July	Aug	Sep	Oct	Total	July		Aug		Sep		Oct		Total	
Mean	16,894.5	10,777.2	7,365.2	1,272.9	36,309.8	5,017.0		3,917.7		2603.9		578.6		12,117.2	
Range	19,502.2	9,481.5	5,074.1	1,511.1	25,694.8	5,464.3		3,429.9		2,155.7		736.6		8,481.7	
SD	3,861.2	2,443.7	1,201.2	326.2	6,303.6	1,248.1		904.3		468.2		168.1		2,021.4	
CV (%)	22.9	22.7	16.3	25.6	17.4	24.9		23.1		18.0		29.0		16.7	
Genetic diversity index															
	2.00	1.94	1.97	1.88		1.87		2.03		1.98		1.86		

SD, standard deviation; CV, coefficient of variation.

WCF-19 (26,044.4 kg/ha) had the highest fresh weight of 56 bermudagrass samples in July, followed by WCF-34 (24,951.1 kg/ha) and WCF-20 (23,911.1 kg/ha). WCF-7 (14,488.9 kg/ha) had the highest fresh weight in August, followed by WCF-5 (14,444.4 kg/ha) and WCF-20 (14,355.6 kg/ha). In September, the fresh weight of WCF-47 (10,222.2 kg/ha) was the highest, followed by WCF-21 (9,555.6 kg/ha) and WCF-5 (9,244.4 kg/ha). Bermudagrass grew slowly in October, with a minimal fresh grass yield. Following cutting, WCF-20 had the greatest fresh weight of 2,177.8 kg/ha, followed by WCF-8 (1,955.6 kg/ha) and WCF-7 (1,933.3 kg/ha). The average total fresh weight of 4 months was 36,309.8 kg/ha; WCF-19 had the greatest total fresh weight, 49,511.1 kg/ha, and Wrangler had the lowest, 23,816.3 kg/ha.

Meanwhile, **Table 2** compared the dry weight variations of 56 bermudagrass. The coefficient of variation of forage grass dry weight ranged between 18.0% and 29.0%. The greatest coefficient of variation of forage grass dry weight in October was 29.0%, while the smallest coefficient of variation of forage grass dry weight in September was 18.0%. WCF-46 (7,536.8 kg/ha) had the greatest dry weight in July, followed by WCF-34 (7,526.4 kg/ha) and WCF-20 (6,835.4 kg/ha). WCF-5 (5,468.7 kg/ha) had the greatest dry weight in August, followed by WCF-20 (5,358.8 kg/ha) and WCF-7 (5,274.4 kg/ha). WCF-7 (3,829.0 kg/ha) had the greatest dry weight in September, followed by WCF-5 (3,611.1 kg/ha) and WCF-24 (3,451.3 kg/ha). WCF-8 (985.9 kg/ha) had the greatest dry weight in October, followed by WCF-13 (938.9 kg/ha) and WCF-20 (929.2 kg/ha). In total dry weight for 4 months, R106 had the lowest dry weight of 7,621.6 kg/ha and WCF-20 had the highest of 16,103.3 kg/ha in 2021.

The genetic diversity index was determined using the fresh weight and dried weight of 56 bermudagrass samples from July to October in 2021. **Table 2** showed that the average value of the genetic diversity index of eight indicators was 1.94, suggesting that 56 bermudagrass materials were diverse in dry and fresh weight.

**Table 3** showed the fresh and dry weight of bermudagrass from June to September 2022. The variation coefficient of the fresh and dry weight of bermudagrass in July was the highest (38.8%, 42.7%). In June, WCF-36 (30,488.9 kg/ha) had the highest fresh weight, followed by WCF-37 (30,266.7 kg/ha) and WCF-34 (29,266.7 kg/ha). In July, WCF-47 (23,888.9 kg/ha^)^ had the highest fresh weight, followed by WCF-48 (23,622.2 kg/ha) and WCF-46 (22,111.1 kg/ha). WCF-47 (11,155.6 kg/ha, 2,000.0 kg/ha) was the highest in August and September of fresh weight. WCF-37 (60,822.2 kg/ha) had the highest total fresh weight in 2022. The total dry weight of WCF-36 (18,127.5 kg/ha) was the highest in 2022. Dried weight was the highest in June of dry weight for WCF-36 (9,264.3 kg/ha). WCF-47 (7,117.5 kg/ha, 3,528.6 kg/ha, 588.1 kg/ha) had the highest dry weight in July, August, and September.

**Table 3:** Comparison of biomass variation of 56 forage bermudagrass samples in 2022.

Items	Fresh weight (kg/ha)					Dry weight (kg/ha)				
	June	July	Aug	Sep	Total	June	July	Aug	Sep	Total
Mean	18,676.3	16,040.4	8,099.9	1,362.0	44,178.7	4,928.5	4,815.5	2,602.8	419.5	12,766.3
Range	26,963.0	17,057.8	6,955.6	1,355.6	35,814.8	8,299.1	5,021.5	2,229.9	399.7	10,984.4
SD	7,252.2	4,161.9	1,351.7	305.2	10,778.1	2,102.6	1,199.7	427.4	95.9	3,132.7
CV (%)	38.8	25.9	16.7	22.4	24.4	42.7	24.9	16.4	22.9	24.5
Genetic diversity index										
	2.03	1.94	2.04	2.04		2.06	2.01	2.02	2.06	

SD, standard deviation; CV, coefficient of variation.

WCF-34 (109,773.3 kg/ha) had the highest total fresh weight for 2 years, WCF-37 (104,577.8 kg/ha) had the second highest total fresh weight for 2 years, WCF-37 (31,951.6 kg/ha) had the highest total dry weight for 2 years, and WCF-27 (31,518.2 kg/ha) had a lower total dry weight than WCF-37 only.

The genetic diversity index was determined using the fresh weight and dried weight of 56 bermudagrass samples from June to September in 2022. **Table 3** showed that the average value of the genetic diversity index of eight indicators was 2.03, suggesting that 56 bermudagrass materials had great genetic diversity.

### 3.2 Genotype-by-month interaction

The fresh weights of R102, R104, R106, R107, R101, R103, R105, and Wrangler were used for the analysis of variance. Two-way ANOVA showed a highly significant effect of genotype as well as month on fresh weight of bermudagrass in 2021 (*p* = 0.013, *p* = 0) and 2022 (*p* = 0, *p* = 0), the interaction of genotype and month had no effect on fresh weight in 2021 (*p* = 0.652), but the interaction of genotype and month had a significant effect on fresh weight in 2022 (*p* = 0.038) (**Table 4**).

**Table 4:** Effects of month, genotype, and their interaction on fresh weight.

	2021			2022		
Source	Free degree	*F* value	*p*	Free degree	*F* value	*p*
Month	3	226.989^**^	0	3	169.948^**^	0
Genotype	7	2.796^**^	0.013	7	6.714^**^	0
Month × Genotype	21	0.849	0.652	21	1.799^*^	0.038

^*^ Significant effect (p< 0.05).

^**^ Extremely significant effect (p< 0.01).

### 3.3 Comparison and analysis of feed quality of bermudagrass

WCF-24 had the lowest Ash level (7.46%), followed by WCF-31 (7.50%) and WCF-38 (7.75%), while WCF-7 had the highest Ash content (14.96%). WCF-26 had the greatest CP content (16.27%), followed by WCF-34 (15.91%) and WCF-25 (15.89%), with WCF-44 having the lowest CP content (10.74%). WCF-27 had the largest CF content (3.58%), followed by WCF-34 (3.24%) and WCF-8 (3.15%). WCF-13 (0.45%) had the greatest total P content, followed by WCF-11 (0.39%) and WCF-39 (0.38%). The lowest NDF was WCF-14 (63.51%), followed by WCF-40 (64.49%) and WCF-42 (65.00%). The lowest ADF was WCF-13 (27.68%), followed by WCF-6 (27.75%) and WCF-35 (27.78%). WCF-42 (95.32) had the greatest RFV, followed by WCF-39 (93.06) and WCF-40 (91.86).

### 3.4 Analysis of feed quality variability and genetic diversity of bermudagrass

**Table 5** showed five nutrition indicators of bermudagrass variation coefficient of between 7.07% and 15.21%: ash (15.21%) > crude fat (12.04%) > P content (11.88%) > crude protein (10.07%) > relative feed value (7.07%). The genetic diversity index of the five indicators was determined based on the nutritional content of 56 bermudagrass samples. As shown in **Table 5**, the genetic diversity index of the five indicators exceeded one, suggesting that the genetic diversity of the 56 bermudagrass samples’ nutrition was high.

**Table 5:** Comparison of feed quality variation in bermudagrass.

Item	Ash	CP	CF	P content	NDF	ADF	RFV
Percentage of dry matter (%)							
Mean	9.27	13.57	2.67	0.32	70.61	33.65	82.81
Range	7.50	5.52	1.69	0.19	15.57	18.24	26.15
SD	1.41	1.37	0.32	0.04	3.98	4.04	5.86
CV (%)	15.21	10.07	12.04	11.88	5.64	12.01	7.07
Genetic diversity index	1.52	2.07	1.93	1.87			2.09

CP, crude protein; CF, crude fat; NDF, neutral detergent fiber; ADF, acid detergent fiber; RFV, relative feed value; SD, standard deviation; CV, coefficient of variation.

### 3.5 Correlation analysis of feed quality

Correlation analysis showed the five quality traits (Ash, CP, CF, P content, and RFV) of 56 bermudagrass samples, and it could be seen in **Table 6** that crude protein was highly significantly and positively correlated with crude fat (*r* = 0.348) among the five traits, with no correlation among other traits.

**Table 6:** Correlation analysis of feed quality in bermudagrass.

	Ash	CP	CF	P content	RFV
Ash	1				
CP	−0.185	1			
CF	−0.152	0.348^**^	1		
P content	0.056	0.080	−0.009	1	
RFV	−0.218	0.129	0.017	0.205	1

CP, crude protein; CF, crude fat; RFV, relative feed value.

^**^ Extremely significant correlation (p< 0.01).

### 3.6 GRA result

According to the screening objectives of bermudagrass varieties, X_0_ was selected by the minimum value of ash, other characters were selected by maximum values (**Table 7**), and the initial value method was used for dimensionless processing to eliminate the differences arising from the order of magnitude of different traits. Calculated from the results of dimensionless processing |X_0_(k) − X*_i_* (k)|, it could be seen that min*_i_* min*_k_* |X_0_(k) − X*_i_* (k)|secondary minimum difference was 0 and max*_i_* max*_k_* |X_0_(k) − X*_i_* (k)|secondary maximum difference was 1.00, using the Equations 1–3 to calculate the 56 bermudagrass materials’ correlation coefficient and weight (**Table 8**). The weighted correlation of bermudagrass was then calculated and sorted using the Equation 4, with the results shown in **Table 9**. According to the results in **Table 9**, WCF-42, WCF-34, WCF-38, WCF-37, and WCF-40 were among the top five highest-ranking, indicating that they were highly correlated with the best bermudagrass and had a good overall performance. WCF-5, WCF-2, WCF-10, WCF-3, and WCF-6 were in the last five ranks, which meant that the correlation with the best bermudagrass was low and the comprehensive performances were poor. The control variety ranked 27, which was in the middle position.

**Table 7:** Main trait indexes of participating materials and ideal varieties.

Number	Ash (%)	CP (%)	CF (%)	P content (%)	RFV	2-year dry weight	Fresh/dry ratio
X_0_	7.46	16.27	3.58	0.45	95.32	31,951.6	3.57
X_1_	9.78	14.05	2.38	0.32	88.41	27,624.7	3.26
X_2_	13.15	12.58	1.89	0.36	82.88	25,450.1	3.23
X_3_	10.71	11.97	1.94	0.28	79.07	25,663.0	3.20
X_4_	10.09	14.51	2.38	0.26	80.30	25,293.9	3.06
X_5_	10.41	12.12	2.34	0.27	76.51	25,773.3	3.30
X_6_	10.58	11.72	2.09	0.35	81.56	22,045.5	3.02
X_7_	14.96	14.04	2.88	0.34	69.17	26,838.4	3.24
X_8_	10.12	13.72	3.15	0.29	74.14	27,536.2	3.21
X_9_	9.49	13.21	2.86	0.30	77.17	25,058.3	3.33
X_10_	14.45	12.64	2.77	0.35	80.81	22,854.1	3.17
X_11_	8.98	12.99	2.67	0.39	89.45	19,140.0	2.85
X_12_	9.30	13.55	3.02	0.38	83.50	19,017.8	3.02
X_13_	9.22	13.30	2.23	0.45	87.52	24,902.7	3.36
X_14_	8.73	15.20	2.93	0.31	89.62	22,070.5	2.98
X_15_	10.19	13.75	2.72	0.29	82.64	25,628.5	3.21
X_16_	8.97	12.20	2.53	0.34	82.77	19,160.7	3.35
X_17_	9.19	14.41	2.33	0.30	89.37	22,436.0	3.23
X_18_	9.29	14.23	2.41	0.27	89.76	21,161.9	3.05
X_19_	10.12	14.75	2.87	0.35	90.27	28,278.2	3.35
X_20_	9.90	14.79	2.89	0.35	85.87	28,175.7	3.30
X_21_	9.42	14.69	2.60	0.38	88.01	25,564.4	3.44
X_22_	9.18	12.10	2.36	0.33	84.19	20,261.5	3.34
X_23_	9.21	12.75	2.64	0.28	85.85	20,183.1	3.15
X_24_	7.46	10.81	2.63	0.32	86.42	24,367.4	2.98
X_25_	8.38	15.89	2.82	0.32	78.85	30,359.2	3.12
X_26_	7.76	16.27	2.23	0.27	72.62	27,385.2	3.44
X_27_	10.05	11.16	3.58	0.28	78.07	31,518.2	2.98
X_28_	8.32	12.96	2.82	0.30	80.75	29,583.4	3.13
X_29_	8.33	15.01	2.86	0.34	72.00	30,272.0	3.27
X_30_	9.15	13.84	2.67	0.28	75.50	26,621.1	3.29
X_31_	7.50	13.57	2.87	0.34	78.44	25,569.1	3.18
X_32_	7.89	15.30	3.06	0.33	84.70	21,891.1	3.13
X_33_	8.99	14.19	2.88	0.29	81.53	27,389.1	3.33
X_34_	8.54	15.91	3.24	0.30	76.80	30,780.9	3.57
X_35_	8.92	13.82	3.08	0.33	89.57	24,214.2	3.37
X_36_	8.30	13.31	2.90	0.32	86.20	29,543.7	3.23
X_37_	9.37	14.86	2.79	0.28	89.72	31,951.6	3.27
X_38_	7.75	15.25	2.74	0.35	87.56	25,961.2	3.41
X_39_	8.72	13.45	2.42	0.38	93.06	25,905.6	3.21
X_40_	8.59	12.97	2.87	0.30	91.86	31,110.2	3.27
X_41_	9.13	15.11	3.02	0.30	84.75	26,884.1	3.28
X_42_	8.50	15.66	3.07	0.30	95.32	27,202.3	3.57
X_43_	8.56	13.01	2.49	0.29	82.15	27,378.8	3.41
X_44_	8.43	10.74	2.35	0.32	79.75	23,064.3	2.98
X_45_	8.60	12.69	2.24	0.27	77.87	28,942.9	3.31
X_46_	9.52	11.34	2.43	0.29	85.26	28,471.8	3.09
X_47_	8.74	11.39	2.38	0.29	80.77	31,276.2	3.21
X_48_	9.32	11.59	2.53	0.29	72.90	28,997.7	3.31
X_49_	8.20	13.07	2.60	0.36	75.03	17,692.8	3.36
X_50_	9.24	12.43	2.70	0.35	85.66	17,327.3	3.30
X_51_	8.70	15.14	2.83	0.37	84.88	14,764.6	3.41
X_52_	8.87	13.49	2.75	0.36	78.63	17,503.7	3.11
X_53_	9.10	13.67	2.77	0.35	75.87	23,509.9	3.23
X_54_	8.35	14.34	2.76	0.34	82.74	19,958.1	3.26
X_55_	8.35	13.42	2.80	0.30	89.31	19,408.7	3.19
X_56_	8.13	14.85	2.68	0.36	83.72	16,552.3	2.98

CP, crude protein; CF, crude fat; RFV, relative feed value.

**Table 8:** The calculated values of grey relational coefficient and weight.

Number	Ash	CP	CF	P content	RFV	2-year dry weight	Fresh/dry ratio
X_1_	0.62	0.79	0.60	0.64	0.87	0.79	0.85
X_2_	0.40	0.69	0.52	0.72	0.79	0.71	0.84
X_3_	0.54	0.66	0.52	0.58	0.75	0.72	0.83
X_4_	0.59	0.82	0.60	0.55	0.76	0.71	0.78
X_5_	0.56	0.66	0.59	0.56	0.72	0.72	0.87
X_6_	0.55	0.64	0.55	0.69	0.78	0.62	0.76
X_7_	0.33	0.79	0.72	0.68	0.65	0.76	0.84
X_8_	0.59	0.76	0.81	0.59	0.69	0.78	0.83
X_9_	0.65	0.73	0.71	0.61	0.73	0.70	0.88
X_10_	0.35	0.69	0.69	0.70	0.77	0.64	0.82
X_11_	0.71	0.71	0.66	0.80	0.89	0.56	0.71
X_12_	0.67	0.75	0.76	0.77	0.80	0.55	0.76
X_13_	0.68	0.73	0.57	1.00	0.86	0.69	0.89
X_14_	0.75	0.88	0.73	0.62	0.89	0.62	0.75
X_15_	0.58	0.76	0.68	0.59	0.79	0.72	0.83
X_16_	0.71	0.67	0.63	0.68	0.79	0.56	0.89
X_17_	0.68	0.81	0.59	0.61	0.89	0.63	0.84
X_18_	0.67	0.80	0.61	0.57	0.90	0.60	0.77
X_19_	0.59	0.84	0.72	0.70	0.90	0.81	0.89
X_20_	0.61	0.85	0.72	0.71	0.84	0.81	0.87
X_21_	0.66	0.84	0.65	0.77	0.87	0.72	0.93
X_22_	0.69	0.66	0.60	0.65	0.81	0.58	0.88
X_23_	0.68	0.70	0.66	0.57	0.83	0.58	0.81
X_24_	1.00	0.60	0.65	0.64	0.84	0.68	0.75
X_25_	0.80	0.96	0.70	0.64	0.74	0.91	0.80
X_26_	0.93	1.00	0.57	0.56	0.68	0.78	0.93
X_27_	0.59	0.62	1.00	0.57	0.74	0.97	0.75
X_28_	0.81	0.71	0.70	0.61	0.77	0.87	0.80
X_29_	0.81	0.87	0.71	0.68	0.67	0.91	0.85
X_30_	0.69	0.77	0.66	0.58	0.71	0.75	0.86
X_31_	0.99	0.75	0.72	0.69	0.74	0.72	0.82
X_32_	0.90	0.89	0.77	0.66	0.82	0.61	0.80
X_33_	0.71	0.80	0.72	0.59	0.78	0.78	0.88
X_34_	0.78	0.96	0.84	0.60	0.72	0.93	1.00
X_35_	0.72	0.77	0.78	0.66	0.89	0.67	0.90
X_36_	0.82	0.73	0.72	0.63	0.84	0.87	0.84
X_37_	0.66	0.85	0.69	0.58	0.90	1.00	0.86
X_38_	0.93	0.89	0.68	0.71	0.86	0.73	0.91
X_39_	0.75	0.74	0.61	0.77	0.95	0.73	0.83
X_40_	0.77	0.71	0.72	0.60	0.93	0.95	0.85
X_41_	0.69	0.88	0.76	0.60	0.82	0.76	0.86
X_42_	0.78	0.93	0.78	0.61	1.00	0.77	1.00
X_43_	0.77	0.71	0.62	0.59	0.78	0.78	0.92
X_44_	0.79	0.60	0.59	0.64	0.75	0.64	0.75
X_45_	0.77	0.70	0.57	0.56	0.73	0.84	0.87
X_46_	0.65	0.62	0.61	0.58	0.83	0.82	0.79
X_47_	0.75	0.63	0.60	0.58	0.77	0.96	0.83
X_48_	0.67	0.64	0.63	0.59	0.68	0.84	0.87
X_49_	0.84	0.72	0.65	0.73	0.70	0.53	0.90
X_50_	0.68	0.68	0.67	0.70	0.83	0.52	0.87
X_51_	0.75	0.88	0.70	0.74	0.82	0.48	0.92
X_52_	0.73	0.75	0.69	0.73	0.74	0.53	0.79
X_53_	0.70	0.76	0.69	0.70	0.71	0.66	0.84
X_54_	0.81	0.81	0.69	0.67	0.79	0.57	0.85
X_55_	0.81	0.74	0.70	0.61	0.89	0.56	0.82
X_56_	0.85	0.85	0.67	0.73	0.80	0.51	0.75
Weight	0.1368	0.1481	0.1308	0.1263	0.1552	0.1393	0.1635

CP, crude protein; CF, crude fat; RFV, relative feed value.

**Table 9:** A total of 56 bermudagrass weighted correlation analysis and ranking.

Number	Correlative degree	Range	Number	Correlative degree	Range
X_42_	0.85	1	X_55_	0.74	29
X_34_	0.84	2	X_47_	0.74	30
X_38_	0.82	3	X_17_	0.73	31
X_37_	0.80	4	X_45_	0.73	32
X_40_	0.80	5	X_49_	0.73	33
X_25_	0.80	6	X_8_	0.73	34
X_29_	0.79	7	X_12_	0.73	35
X_19_	0.79	8	X_53_	0.73	36
X_26_	0.79	9	X_11_	0.72	37
X_36_	0.78	10	X_30_	0.72	38
X_32_	0.78	11	X_9_	0.72	39
X_21_	0.78	12	X_15_	0.71	40
X_13_	0.78	13	X_50_	0.71	41
X_35_	0.78	14	X_18_	0.71	42
X_20_	0.78	15	X_16_	0.71	43
X_31_	0.78	16	X_52_	0.71	44
X_39_	0.77	17	X_48_	0.71	45
X_41_	0.77	18	X_46_	0.71	46
X_51_	0.76	19	X_22_	0.70	47
X_28_	0.76	20	X_23_	0.70	48
X_33_	0.76	21	X_4_	0.69	49
X_14_	0.75	22	X_7_	0.69	50
X_27_	0.75	23	X_44_	0.68	51
X_43_	0.75	24	X_5_	0.68	52
X_54_	0.75	25	X_2_	0.67	53
X_1_	0.74	26	X_10_	0.67	54
X_56_	0.74	27	X_3_	0.66	55
X_24_	0.74	28	X_6_	0.66	56

### 3.7 Cluster analysis of feed quality

**Figure 1:**
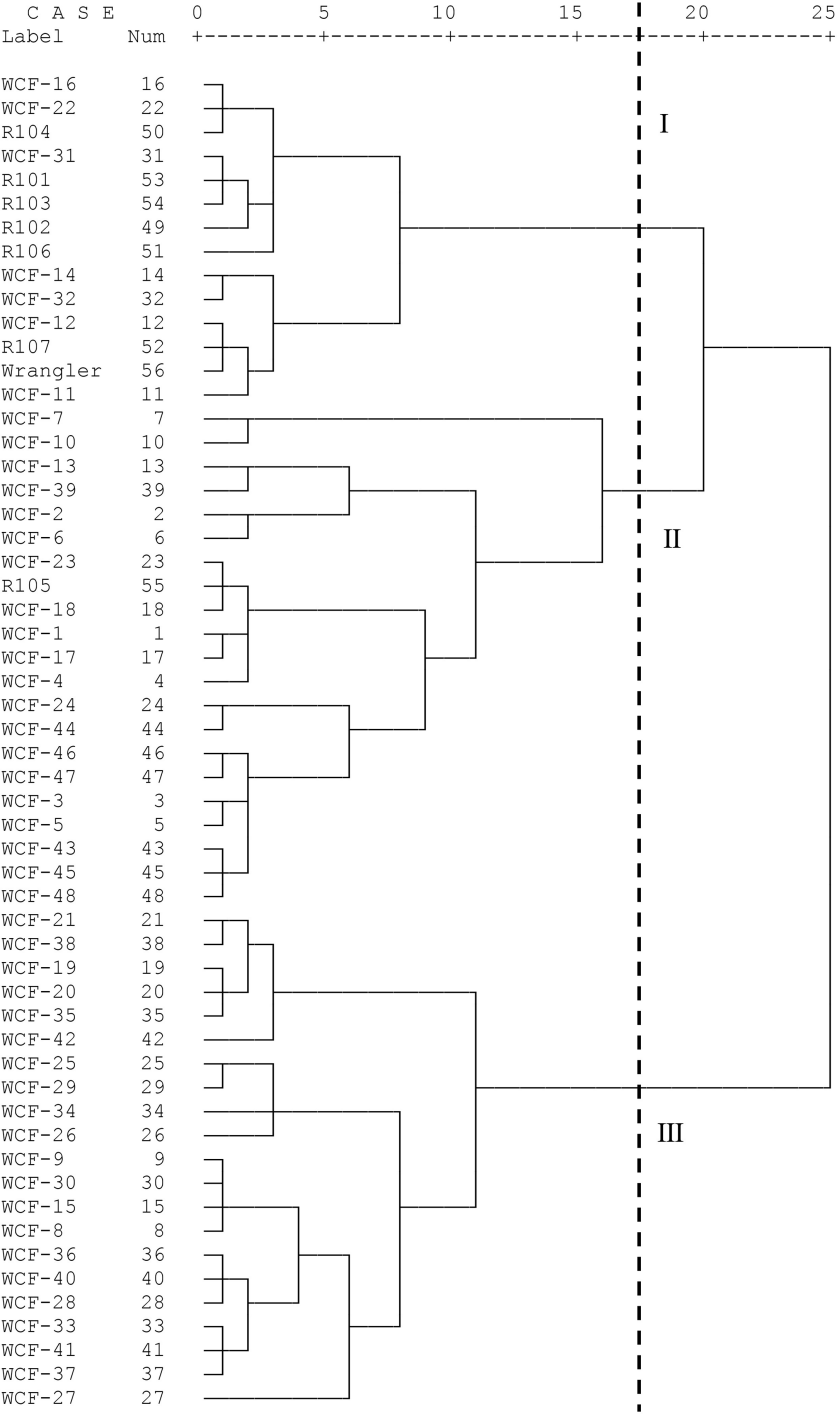
Cluster analysis of 56 bermudagrass samples.

Seven traits (Ash, CP, CF, P content, RFV, 2-year dry weight, and fresh/dry ratio) were selected as evaluation indexes. After standardizing the data, the Euclidean distance was computed, and SPSS 16.0 was used to perform Ward’s method cluster analysis on 56 bermudagrass. Bermudagrass was classified into three groups (**Figure 1**).

Cluster analysis divided the 56 bermudagrass samples into three groups, of which group 2 was significantly different from group 1 and group 3 on Ash (*p* = 0.015), group 1 and group 3 were significantly different from group 2 on CP (*p* = 0) and CF (*p* = 0), and group 1 was significantly different from group 2 and group 3 on P content (*p* = 0.003). There was no significant difference in RFV among the three groups. There was a significant difference in total dry weight over 2 years among the three groups (*p* = 0). Group 3 was significantly different from group 1 and group 2 on fresh/dry ratio (*p* = 0.015). According to **Table 10**, most indicators of group 3 were higher than those of the other groups, and group 3 had a better overall quality.

**Table 10:** Comparison of feed quality among different groups.

Item	Group		
	Group 1	Group 2	Group 3
Ash(%DM)	8.65 ± 0.55b	9.94 ± 1.97a	9.02 ± 0.77b
CP/(%DM)	13.71 ± 1.11a	12.70 ± 1.18b	14.34 ± 1.23a
CF/(%DM)	2.75 ± 0.19a	2.41 ± 0.25b	2.89 ± 0.26a
P content/(%DM)	0.35 ± 0.02a	0.31 ± 0.04b	0.31 ± 0.03b
RFV	82.80 ± 4.43a	82.80 ± 5.95a	82.82 ± 6.79a
2-year dry weight	19,601.4 ± 2,901.5c	25,144.8 ± 3,113.4b	28,143.7 ± 2,281.9a
Fresh/dry ratio	3.18 ± 0.17b	3.19 ± 0.12b	3.30 ± 0.14a

Different lowercase letters indicate significant differences between the different groups at the 0.05 level.

The top five GRA-ranked bermudagrass samples were all in group 3, so the results of GRA were combined to select five superior bermudagrass from 56 bermudagrass samples for WCF-42, WCF-34, WCF-38, WCF-37, and WCF-40.

## 4 Discussion

The genetic diversity index between dry and fresh weights in 4 months was substantial, showing that 56 bermudagrass samples had rich genetic variety in dry and fresh weight indicators. The stronger the coefficient of variation, the greater the difference between the bermudagrass. The coefficient of variation of bermudagrass dry weight was the greatest in October. This might be because different genetic factors in bermudagrass lead to different tolerances to low temperatures, resulting in large differences in dry weights. The yields of bermudagrass declined progressively as the temperature fell. In the first year, the highest average fresh weight was obtained in July and the lowest average yield in October; in the second year, the highest average fresh weight was obtained in June, and the lowest average fresh weight was obtained in September. The yield of coastal bermudagrass showed a downward trend in late summer and early autumn, which was consistent with the trend of gradual decline in production in this experiment (Burton et al., 1988). The fresh weights of bermudagrass were regulated by the months as well as genetic variables. The results of the ANOVA indicated that genotype and month affected the fresh weight of bermudagrass, and similar results were found in the study of maize yield (Fernando Hernández et al., 2014), There was no interaction between genotype and month in 2021, while there was an interaction between genotype and month in 2022; the reason for this phenomenon may be due to the different climatic conditions in these 2 years (Wachira et al., 2002).

There had been many studies on the genetic variability of bermudagrass phenotypic features, but few on the genetic diversity of feed quality. The study of genetic variability in bermudagrass feed quality was critical for the improvement of kinds of good bermudagrass. The coefficient of variation of coarse ash in 56 bermudagrass samples was the highest, but the genetic diversity index was the lowest, indicating that the highest coefficient of variation was not always the highest genetic diversity index. The crude protein concentration and crude fat had a substantial relationship. This was consistent with the findings of Duan et al. (2017) on cereals, but not with Huang et al. (2011). This might be owing to variances in bermudagrass varieties. The genetic variety index of crude protein content in this test was only slightly lower than the relative feed value, reaching 2.1, showing that bermudagrass had a high genetic diversity of crude protein content. According to Peng et al. (2017), the protein content in bermudagrass “Anza 1” at a height of 40 cm was 13.1%, and the average protein value of bermudagrass in this test was 13.57%, which was higher than that of “Anza 1”, indicating that this bermudagrass population could be used to screen high protein forage bermudagrass. The genetic diversity index of the crude fat of bermudagrass in this test was 1.9, indicating that the genetic diversity of the crude fat of bermudagrass was rich. The average value of the crude fat of 56 samples was 2.67%, which was lower than the crude fat determined by Zhang (2020), indicating that this population of bermudagrass was not conducive to the screening of high-fat-content forage grass, and the reason for this result might be the late sampling time of bermudagrass. The fiber level of the grass would alter its palatability and digestibility (Xie et al., 2021). According to the American grassland quality standard, RFV was classified as Level 1 at 125–151, Level 2 at 103–124, and Level 3 at 87–102 (Peng et al., 2019). The RFV values of 56 test materials were less than 100, with WCF-42 being the highest (95.32). It could be shown that 56 bermudagrass materials fulfill the Level 3 criterion, and the average amount of acid detergent fiber was higher than that of Tifton85 bermudagrass measured by Yang et al. (2022), The amount of neutral detergent fiber in Tifton85 bermudagrass was lower, and this difference was due to herbage variety, as a herbage’s nutritional content changes with growing stage. This experiment was carried out in July; at the moment, bermudagrass had a high protein level but a low neutral detergent fiber and acid detergent fiber concentration.

The yield and the content of nutritional value were important indicators to evaluate the quality of bermudagrass. GRA could make the results of a comprehensive analysis of multiple characters objective and fair and had been applied to feed crops such as soybean and alfalfa as well as bermudagrass for drought resistance evaluation (Zeng et al., 2020; Wang et al., 2022). In this study, we selected the optimal value of each trait as the baseline to calculate the weight of each trait for sorting. The resulting order was as follows: fresh/dry ratio (0.1635) > RFV (0.1552) > CP (0.1481) > 2-year dry weight (0.1393) > Ash (0.1368) > CF (0.1308) > P content (0.1263). Tian et al. (2014) made a comprehensive evaluation of the nutritional value of wild grass herbage, and the weight coefficients of each character index were obtained according to the grey correlation degree ranked as CP > crude fiber > CF > Ash > P content, which was similar to this study. A similar result was also obtained on alfalfa (Ma et al., 2022). In this study, we then calculated and ranked the weighted correlation coefficient of each bermudagrass and finally obtained the top five bermudagrass: WCF-42, WCF-34, WCF-38, WCF-37, and WCF-40.

Currently, cluster analysis was utilized to explore the genetic diversity of many species. In this paper, the indicators used for cluster analysis were based on Huang et al. (2011), and the yield index was added on this basis. The results of cluster analysis divided 56 bermudagrass samples into three populations that exhibit differences in Ash, CP, CF, P content, 2-year dry weight, and fresh/dry ratio, while Deng et al. (2014) and Guo et al. (2021) produced different classification results based on CP and fiber. This difference could be caused by different indicators used by researchers.

## 5 Conclusion

This study investigated the yield traits of bermudagrass samples from 2 years and the quality traits from 1 year. The results showed that the agronomic traits of 56 bermudagrass samples had rich genetic diversity. In terms of yield, WCF-34 and WCF-37 showed great potential in fresh weight and dry weight, respectively. In terms of quality, the high-protein line WCF-26 and the high-RFV line WCF-42 could be used as experimental materials to improve the RFV quality of bermudagrass crude protein. Through comprehensive analysis of the agronomic characters of bermudagrass, five bermudagrass samples—WCF-42, WCF-34, WCF-38, WCF-37, and WCF-40—were selected for displaying better performance than Wrangler. These five types of bermudagrass had great potential as high-quality feed and could also be used as experimental materials for bermudagrass variety improvement. This study would provide a theoretical basis for the improvement of bermudagrass varieties.

## Data availability statement

The original contributions presented in the study are included in the article/supplementary material. Further inquiries can be directed to the corresponding author.

## Author contributions

JF contributed to the conception and design of the study. JC, SL, XC, MJ, and LX performed the statistical analysis. JC wrote the first draft of the manuscript. SF and EA wrote sections of the manuscript. All authors contributed to the article and approved the submitted version.
